# Targeting Metabolic Remodeling in Glioblastoma Multiforme

**DOI:** 10.18632/oncotarget.190

**Published:** 2010-10-27

**Authors:** Amparo Wolf, Sameer Agnihotri, Abhijit Guha

**Affiliations:** ^1^ The Arthur and Sonia Labatt Brain Tumor Research Centre, Hospital for Sick Children Research Institute, University of Toronto, Toronto, Ontario, Canada, M5G 1L7; ^2^ Division of Neurosurgery, Toronto Western Hospital, University of Toronto, Toronto, Ontario, Canada, M5T 2S8

**Keywords:** Tumor Metabolism, Aerobic Glycolysis, Warburg Effect, Glioblastoma Multiforme

## Abstract

A key aberrant biological difference between tumor cells and normal differentiated cells is altered metabolism, whereby cancer cells acquire a number of stable genetic and epigenetic alterations to retain proliferation, survive under unfavorable microenvironments and invade into surrounding tissues. A classic biochemical adaptation is the metabolic shift to aerobic glycolysis rather than mitochondrial oxidative phosphorylation, regardless of oxygen availability, a phenomenon termed the “Warburg Effect”. Aerobic glycolysis, characterized by high glucose uptake, low oxygen consumption and elevated production of lactate, is associated with a survival advantage as well as the generation of substrates such as fatty acids, amino acids and nucleotides necessary in rapidly proliferating cells. This review discusses the role of key metabolic enzymes and their association with aerobic glycolysis in Glioblastoma Multiforme (GBM), an aggressive, highly glycolytic and deadly brain tumor. Targeting key metabolic enzymes involved in modulating the “Warburg Effect” may provide a novel therapeutic approach either singularly or in combination with existing therapies in GBMs.

## INTRODUCTION

Metabolic remodeling is a predominant phenotype in cancer cells and refers to the alterations in the utilization and/or synthesis of important metabolites including glucose, glycogen, fatty acids, amino acids, and glutamine by tumor cells. In the early 1920s, Otto Warburg was the first to describe a metabolic adaptation that occurs within solid tumors, for which he ultimately received a Nobel Prize [1]. Warburg noticed that normal tissues generally use glycolysis to generate about 10% of the total cellular ATP with the mitochondria generating the remaining 90%, the latter involving a process termed oxidative phosphorylation (OXPHOS). However, in many tumor tissues, more than 50% of cellular ATP can be generated from glycolysis, even in the presence of oxygen, thus termed aerobic glycolysis. By using aerobic glycolysis (or “Warburg Effect”) even in the presence of oxygen and functional mitochondria, tumor cells divert more of the generated pyruvate to extra-mitochondrial breakdown to lactate, the product of anaerobic glycolysis. There are several advantages for this switch to aerobic glycolysis by tumor cells, as discussed below. In addition, the increased aerobic glycolysis in cancer is exploited for diagnostic purposes. For example, Positron Emission Tomography (PET) takes advantage of the increased glucose uptake by using 2-Deoxyglucose labeled with ^18^F radioemitter, to distinguish regions of tumor from non-tumor or necrotic regions. Non-invasive MR techniques such as H-MR spectroscopy (MRS) of the brain also takes advantage of this increased aerobic glycolysis by gliomas, measuring increases in choline and lactate levels with reduction of N-acetyl aspartate, the latter associated with normal neurons. In fact, the MRS profile correlates to grade of gliomas, with the most poorly and unfortunately the most common Grade IV glioma (also known as Glioblastoma Multiforme, GBM) having the highest levels of choline and lactate [2,3].

## ALTERED GLUCOSE METABOLISM IN GLIOBLASTOMA MULTIFORME

GBMs are the most common primary human malignant brain tumor, highly therapeutically resistant and have a median survival of 12–16 months despite surgery, radiation and chemotherapy. GBMs are characterized by pathological heterogeneity with regions of pseudopalisading perinecrotic cells under moderate levels of hypoxia (pO2 = 2.5–5%) and infiltrating tumor cells into normal brain under normal oxygen conditions (pO2 = 10%) [4]. GBMs upregulate glycolysis more than three times that of normal brain tissue, measured by increased lactate:pyruvate ratio [5]. Using stereotactic microdialysis of tumor and normal brain in GBM patients the ratio of lactate:pyruvate was higher in tumor tissue compared to adjacent brain [6].

Several in vitro studies have demonstrated large variability in mitochondrial respiration and glucose dependency in cell lines derived from GBM tissues and xenografts [7-9]. The ability to modulate mitochondrial respiration is an important component of tumor cell survival under hypoxic conditions [10-12]. Although not specific to GBMs, considerable evidence also exists supporting metabolic cross-talk between stromal and tumor cells. For example, lactate generated from stromal cells can be taken up by tumor cells, which over-express lactate transporters, and be used as fuel for metabolism [13]. In addition, lactate produced by anaerobic glycolysis in stromal and tumor cells within hypoxic regions can be used as an oxidative substrate by tumor cells in adjacent regions where oxygen supply is greater [8,14]. In addition to enhanced glucose uptake and aerobic glycolysis, GBM cells also exhibit altered glutamine catabolism, particularly within myc expressing cells [15,16]. How increased glutaminolysis and aerobic glycolysis contribute to metabolic remodeling and provide growth and survival advantage, is discussed below.

## PUTATIVE ADVANTAGES OF METABOLIC REMODELING IN GBMS

It was originally hypothesized that the predominant advantage of the Warburg Effect was enhanced glycolytic flux resulting in significantly faster production of ATP per mole of glucose. This would ensure that ATP levels met the demands of highly proliferating tumor cells, particularly in an hypoxic environment. However, evidence suggests that no matter the extent of cell division, tumor cells maintain high ATP to ADP ratios as well as NADH/NAD+, supporting that ATP is never limiting in these cells [17,18]. Furthermore, under non-stressful conditions when resources are not scarce, ATP would be more efficiently provided by OXPHOS. This suggests other growth and survival advantages exist for tumor cell metabolic remodeling.

### Substrates for biosynthetic/anabolic pathways

**Figure 1:**
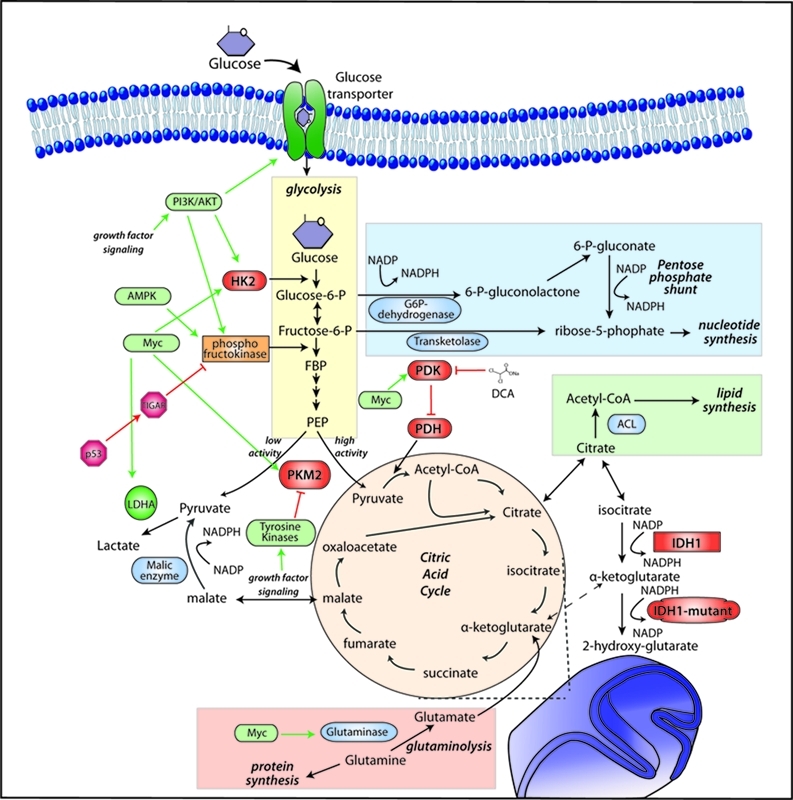
Schematic of Metabolic Remodeling in GBM Enzymes of glycolysis, the pentose phosphate pathway, fatty acid and glutamine metabolism and their regulation by known oncogenes and tumor suppressor genes in proliferating cells. Growth factor/PI3K/AKT signaling stimulates glucose uptake and flux through the early part of glycolysis. Tyrosine kinase signaling negatively regulates flux through at PKM2, making glycolytic intermediates available for macromolecular synthesis. Myc has been found to promote glutamine metabolism and inhibit oxidative metabolism by activating PDK. p53 decreases metabolic flux through glycolysis in response to cell stress.

In order for tumor cells to proliferate, they require nucleotides, fatty acids, membrane lipids, and proteins in addition to ATP. Therefore, alterations in tumor cell metabolism assist in the synthesis of biosynthetic precursors [19] to make these macromolecules. Increased glutaminolysis and accelerated aerobic glycolysis provides a constant supply of metabolic intermediates necessary for macromolecule biosynthesis that facilitates cell growth and proliferation [20]. Two major biosynthetic activities required by proliferating cells is the production of ribose-5-phosphate (R5P) for nucleotide biosynthesis and fatty acids for lipid synthesis (Figure 1).

To generate R5P towards nucleic acid synthesis, tumor cells divert carbon from glycolysis into the oxidative or non-oxidative arm of the pentose phosphate pathway (PPP). Tight regulation at different steps of glycolysis, including the enzymes phosphofructokinase 1 (PFK1 by TIGAR), phosphoglycerate mutase (by p53) and pyruvate kinase M2 (by growth factor signaling), can result in the accumulation of substrates and diversion of carbon towards R5P. The non-oxidative arm of PPP seems to be more important for tumor cells based on higher expression and activity of transketolase (TKTL1) found to correlate with rate of tumor growth in some cancers including GBMs [21,22]. A further level of regulation for R5P synthesis is the ratio of NADP+:NADPH in cells through the oxidative arm at the initial step of PPP. The reversible reduction of glucose-6-phosphase (G6P) by G6P dehydrogenase is associated with reduction of NADP to NADPH, an important reducing agent for several reactions including fatty acid synthesis.

Tumor cells also employ glucose to assist in the generation of fatty acids (Figure 1). Tumor cells require fatty acids to alter membrane-targeted proteins in cell signaling and for *de novo* membrane synthesis. Towards the importance of lipid synthesis, perturbations in enzymes involved in lipid synthesis inhibit tumorigenesis. For example, depletion of ATP citrate lyase (ACL), the enzyme that converts citrate into the lipid precursor cytosolic acetyl-coA, results in decreased GBM growth [23]. Acetyl-coA carboxylase (ACC) and fatty acid synthase (FAS) are upregulated in many cancers and also play a role in tumorigenesis [24,25]. FAS synthesizes long chain fatty acids (e.g. palmitate) from malonyl-coA and NAPDH. Regulation by signaling pathways, such as the PI3K/AKT/mTOR pathway also modulate lipid synthesis. Activation of this pathway increases nuclear localization of the transcription factor sterol response element binding protein-1 (SREBP-1), which then induces expression of lipogenic genes like ACL, ACC and FAS [26].

In addition to enhanced glucose uptake and growth factor signaling, fatty acid synthesis also requires two supporting pathways: the generation of NADPH and anaplerosis. A large amount of NAPDH is required for the synthesis of fatty acids (e.g. the 16-carbon fatty acid palmitate requires 14 molecules of NAPDH). Anaplerotic reactions are those that form intermediates of the TCA cycle, which require replenishing when extracted for biosynthesis. For example, the export of mitochondrial citrate for fatty acid synthesis requires the replacement of oxaloacetate (OAA) by anaplerosis to replenish the exported citrate. Glutaminolysis is an important metabolic pathway in tumor cells, which can augment generation of NAPDH and also replenish TCA intermediates (anaplerosis), as demonstrated in Figure 1. The rate of consumption of glutamine by tumor cells is much higher than required for protein synthesis [27]. The first step of glutaminolysis is the deamidation of glutamine to glutamate via the enzyme glutaminase (GLS), and then the conversion of glutamate to α-ketoglutarate (α-KG) via glutamate dehydrogenase. Glutamine-derived α-KG is a TCA intermediate and the major source of OAA, which replenishes the exported citrate for fatty acid synthesis, in some tumor cells. For example, 13C labeled NMR spectroscopy in human GBM cells demonstrate glutamine to contribute to the bulk of anaplerotic carbon to the TCA cycle [15,28]. In addition, glutaminolysis also results in export of glutamine-derived malate for NAPDH production by cytoplasmic malic enzyme (*ME1* gene) which converts malate to pyruvate and is also a SREBP-1 target. In summary, glutamine metabolism as an anaplerotic precursor and source of NAPDH results in the secretion of a large quantity of glutamine-derived carbon and nitrogen as lactate and alanine. There are other potential sources of NAPDH including G6PD from the oxidative arm of PPP and cytosolic IDH1 and mitochondrial IDH2. The relative importance of these metabolic pathways and their associated enzymes in tumor remodeling and tumor growth is currently unclear and under study.

### Cell survival

In addition to providing substrates for biosynthetic reactions, aerobic glycolysis provides a cell survival advantage to tumor cells through several different, yet interrelated mechanisms. This metabolic remodeling to aerobic glycolysis facilitates adaptation of tumor cells to conditions of fluctuating oxygen levels resulting from altered vascularity [29]. First, the glycolytic phenotype is associated with enhanced mitochondrial membrane polarization (i.e. hyperpolarized) and resistance to mitochondrial membrane permeability [30,31]. Glycolytic enzymes directly regulate the mitochondrial permeability transition pore (PTP) by interacting with the voltage dependent anion channel (VDAC). For example, the glycolytic enzyme Hexokinase 2 (HK2) can localize to the outer mitochondrial membrane and interact with the mitochondrial PTP, in response to Akt pathway activation, thereby regulating the intrinsic apoptotic pathway [32,33]. Our experiments demonstrate this also to occur in GBMs, which aberrantly overexpress HK2, compared to lower grade gliomas or normal brain. Complete or even partial loss of OXPHOS by metabolic remodeling of tumor cells to aerobic glycolysis can also induce resistance to apoptosis by suppressing the activation of pro-apoptotic proteins Bax and Bak, important mediators of mitochondrial membrane permeability (MMP) [34].

Second, the generation of reactive oxygen species (ROS), which promote apoptosis, is decreased by tumor cell remodeling to aerobic glycolysis. The resultant decreased ROS and generation of lactate by aerobic glycolysis protects tumor cells from oxidative stress [35,36]. Other mechanisms of modulating ROS levels include loss of p53 regulation of the enzyme TIGAR, resulting in enhanced apoptosis likely due to increases in ROS [37]. Additionally, enhanced formation of G6P results in greater flux into the PPP to generate NAPDH, which reinforces the tumor cells antioxidant defenses and protects them from ROS mediated apoptosis [37,38].

Furthermore, by favouring aerobic glycolysis, tumor cells generate bicarbonic and lactic acids. These are exported from the cell and render the extracellular environment more acidic, potentially favouring tumor invasion by pH-dependent activation of cathepsins and metalloproteinases [39] and suppressing anti-cancer immune effectors such as T cells [40].

## METABOLIC PATHWAYS UP- AND DOWN-REGULATED IN GBMS: THE CANCER GENOME ATLAS

The Cancer Genome Atlas (TCGA) has characterized a large cohort of human GBM tumors with respect to transcript and microRNA expression, chromosomal copy number, loss of heterozygosity (LOH), methylation, and sequencing. Early reports from the TCGA reinforce previous knowledge of the importance of deregulation of Rb, p53 and receptor tyrosine kinase (RTK)/PI3K/Ras pathways in GBMs [41]. The large-scale analysis also highlighted the importance of *NF1* deletions and inactivation mutations of *PIK3CA* among others in GBM.

The array files from TCGA batch 8 (HG-U133A Affymetrix Array platform) were mined to look at differentially up- and down-regulated metabolism associated genes relative to normal brain with respect to: 1) glucose metabolism 2) fatty acid metablism 3) nucleotide metabolism 4) glutamine metabolism, and evaluated using Ingenuity Pathway Analysis (IPA). Table 1 depicts a subset of significantly up- and down-regulated genes relating to these four metabolic processes according to IPA. For enzymes related to glycolysis, Hexokinase 2 (HK2) was significantly up-regulated while normal brain HK1 is down-regulated. Isocitrate dehydrogenase 1 (IDH1) transcript levels are also elevated while mitochondrial IDH3a are down-regulated. Of interest, several of the enzymes required for glutaminolysis are down-regulated in GBMs including glutaminase (GLS) and glutamate dehydrogenase 1 and 2 (GLUD1,2). The role of many of these differentially expressed metabolic related genes in GBM growth and survival remains to be discovered.

**Table 1:** Differentially expressed metabolic genes in GBMs at the RNA level GBM expression array data was obtained from TCGA (http://tcgadata.nci.nih.gov/tcga/dataAccessMatrix.htm?mode=ApplyFilter&showMatrix=false). In Brief, array files from TCGA batch 8 (HG-U133A Affymetrix Array platform) which contains 25 GBM samples (group1) and 10 Normal brain samples (group2) were imported into Affymetrix Gene Expression Console. Normal brain and GBMs were compared against each other for differential gene expression using significance analysis of microarrays (SAM) (False Discovery Rate of 10% and a minimum fold change of 2). Genes of various metabolic processes from the significantly identified gene list were extracted using Ingenuity Pathway Analysis. This analysis does not discern differentially expressed genes at the protein level, protein modifications or mutations. See Supplemental Table for TCGA sample ID's used in this review.

Up-regulated		Down-regulated	
Primary Metabolic Process	Gene	Primary Metabolic Process	Gene
Glucose Metabolism	FABP5 HK2 ITGB1 MSTN MYC PRKAA1 ZBTB20 IDH1	Glucose Metabolism	ALDH5A1 BDNF CACNA1A NISCH NPY1R PDK2 PDK4 HK1 IDH3a
Fatty Acid Metabolism	ACOT9 ALOX5 ALOX15B ALOX5AP CAV1 CD36 CD74 CEBPB CROT DBI EDN2 ELOVL2 FABP4 FCER1A GGT5 LPL PDPN PECI PLA2G5 PTGS1 QKI SLC27A3 SUCLG2 SYK TNFRSF1 AT NXB	Fatty Acid Metabolism	AACS ACOT7 ACSL6 AKR1C2 ALDH5A1 ANGPTL3 ANKRD26 FAAH HNF4A MYO5A OXCT1 PDK4 PLP1 PRKAR2B SCD SLC27A2 SNCA
Glutamine Metabolism	DDAH2 GFPT2 MECP2	Glutamine Metabolism	ALDH5A1 GLS GLUD1 GLUD2
Nucleotide Metabolism	ADA REXO2 TYMP UPP1	Nucleotide Metabolism	CSGALNACT1 ENTPD3 UGP2

## THE MOLECULAR BASIS OF METABOLIC REMODELING: THE ROLE OF METABOLIC ENZYMES IN GBM

The molecular basis of altered metabolism is likely to be multi-factorial and cancer type dependent. Several reviews have been published detailing the role of the tumor microenvironment and stabilization of HIF1α [42], growth factor/PI3K/AKT [43], and p53 [44] in metabolic remodeling. However, specific metabolic enzymes have been implicated downstream in establishing the Warburg effect. Primary mutations, altered isoform expression profile, and altered regulation/function secondary to oncogenic signaling pathways or the tumor microenvironment are potential mechanisms resulting in the deregulation of metabolic enzymes with ensuing metabolic modification.

### Mutant IDH1

Altered metabolism has the potential of both 1) initiating carcinogenesis and 2) being crucial for the progression of cancer. Mutations of the IDH metabolic enzyme family is very frequent and an early genetic alteration in lower grade astrocytomas and oligodendrogliomas [45,46]. Other than gliomas, mutations in IDH1 have only been reported in Acute Myelogenous Leukemia (AML) [47], suggesting a potential role in tumor initiation. This mutation was identified at residue R132 in IDH1 (and analogous R172 in IDH2) located within the isocitrate (substrate) binding site, and is commonly mutated to histidine (R172H). The somatic mutation of R132 residue occurs in greater than 80% of lower grade II and III astrocytomas and oligodendrogliomas as well as secondary GBMs that develop from these lower grade lesions [45,46,48-51]. Mutation analysis of IDH2, which are much rarer, revealed somatic mutations at the analogous R172IDH2 residue, with most mutations occurring in tumors that lacked *IDH1* mutations [46,48]. Of interest, TCGA analysis has grouped GBMs into four subtypes including classical, mesenchymal, proneural and neural, with some associated molecular characteristics and clinical prognostics to the groups. *IDH1* mutations were predominantly associated with the proneural subtype, which does have a better overall prognosis [52].

Watanabe and colleagues reported that *IDH1* mutations always preceded acquisition of *TP53* mutations (in astrocytomas) and loss of 1p/19q (in oligodendrogliomas) [51]. These results suggest that IDH mutations are early genetic events in a cell of origin that gives rise to both astrocytes and oligodendrocytes. Clinically, GBM patients with *IDH* mutations are significantly younger and have a median overall survival of 31 months compared to 15 months in patients with wild-type *IDH* [46]. Multivariate analysis confirmed that *IDH1* mutations are an independent favourable prognostic indicator even when adjusting for age, MGMT status, and treatment [53]. As a whole, it appears that IDH1 and IDH2 mutations may define a specific subtype of disease with a specific pattern of genetic mutations (e.g. TP53) not seen in primary GBMs (e.g. *PTEN, EGFR, CDKN2A/CDKN2B*). This data has to be taken in the context of the well-known fact that secondary GBMs have a better prognosis than the primary GBMs, which are most prevalent and hence by itself does not provide clear evidence that *IDH1* mutation is somehow involved in more favourable GBM biology. Of interest, *IDH* mutations have not been reported in pediatric GBMs, furthering the thesis that pediatric GBMs, which are much more rare but relatively a common and lethal pediatric CNS tumor, arises from different molecular pathways than adults. Much research is required for our understanding of the molecular biology of these pediatric GBMs, as they do not respond to therapies which have shown some efficacy in adults.

*IDH1* mutations occur in only a single allele (i.e. heterozygotic), suggesting the mutation acts in an oncogenic manner rather than being part of a typical tumor suppressor mutation to promote tumorgenesis. The mechanism(s) of this hypothesized oncogenic activity of the mutation is currently not well-known and subject of much research. First, *IDH1* mutation impairs the ability of IDH1 to convert isocitrate to KG [46,54] and results in decreased generation of αKG and subsequent stabilization of HIF1α [54], which is well known to promote angiogenesis and invasion. Second, *IDH1* mutations result in the neomorphic enzyme catalyzing the NADPH-dependent reduction of αKG to R(-)-2-hydroxyglutarate (2HG) [55]. 2-HG metabolite levels are elevated in glioma specimens [55], with increased 2HG having several potential effects on tumor cells: 1) increased ROS levels due to decreased NADPH, 2) toxicity from competitively inhibiting αKG and glutamate using enzymes including a) transaminases necessary for utilization of glutamate nitrogen for amino and nucleic acid biosynthesis, b) prolyl hydroxylases that regulate HIF1α. To date, it remains unclear how 2HG accumulation promotes the Warburg effect in GBMs, if at all. Elevated 2HG levels in tumor tissues with associated sera may be a potential biomarker to help identify GBM patients with *IDH1* mutations, but to date this has not proven true in GBMs, unlike AML patients sera, which of course is markedly different as a liquid rather than solid cancer.

Of great interest is the observation that production of 2HG does not solely arise from mutations of IDH1 and IDH2. Patients suffering from hereditary Hydroxyglutaric aciduria have loss of function in genes involved in the oxidation of 2HG to αKG. These patients are deficient for either L-2-hydroxyglutarate dehydrogenase (L2HGDH) or R-2-hydroxyglutarate dehydrogenase (R2HGDH). Patients deficient in L2HGDH carry an increased risk for developing brain tumours [56]. Using this as a rationale to explain alternate methods of 2HG production, a recent study examined secondary GBMs and other CNS tumours for inactivating mutations of D2HGDH or L2HGDH. This study was unable to identify any somatic mutations, tumour associated polymorphisms or epigenetic silencing of D2HGDH or L2HGDH [57] in patients lacking IDH1 or IDH2 mutations [57]. Originally identified as somatic mutations, a separate study has detected heterozygous germline mutations in IDH2 that alter enzyme residue R140 in 15 unrelated patients with d-2-hydroxyglutaric aciduria. This may support recent lines of evidence that IDH1/2 mutations may be causal of tumour initiation in germline syndromes such as hydroxyglutaric aciduria [58]. The impact of IDH1/2 mutations on metabolic remodeling and aerobic glycolysis remains an avenue of intense investigation.

### Hexokinase 2

If IDH1 mutations play an important role in the Warburg effect and metabolic remodeling, the clear evidence for which is lacking, this would still only explain less than 10–15% of all GBMs, implying a role for other regulators. An important role of the glycolytic enzyme HK2 was first demonstrated in hepatomas in which it was shown that the glucokinase isoform expressed in liver is substituted by HK2 [59]. Similarly, several reports including TCGA dataset support up-regulation of HK2 in GBMs [60] and our own work (Wolf et al., under review), with variable levels of HK1 expressed in normal brain. As the first enzyme of the glycolytic pathway, HK controls glucose flux into glycolysis or the PPP. HK2 is a highly regulated form of hexokinase, whose transcript is regulated by HIF1α, glucose, insulin, glucagon, cAMP, p53, among others [61]. Mitochondrial binding of HK2 to the outer membrane promotes its stability and reduces feedback inhibition from its product G6P [62]. HK2 interacts with VDAC at the mitochondria and regulates the release of cytochrome *c* and intrinsic apoptosis, although the exact mechanisms of this association are not well understood [32,63].

Our data supports that HK2 plays an important role in establishing the glycolytic phenotype in GBMs. We have shown that stable depletion of HK2 in GBM cells inhibits aerobic glycolysis and promotes normal oxidative glucose metabolism, reflected by decreased extracellular lactate, increased expression of OXPHOS proteins and increased O_2_ consumption (Wolf et al, under review). Associated with a return in oxidative glucose metabolism is a sensitization to cell death inducers including radiation and temozolomide. Over-expression of HK2 in GBM cells promotes proliferation and lactate formation and is dependent on both its mitochondrial localization and kinase activity. The addition of the isoform HK1 to cells depleted of HK2 does not rescue the aerobic glycolytic phenotype despite a return in total hexokinase activity, supporting a unique role of HK2 over HK1 in GBM growth. Reduction of HK2 expression in GBM cells has a pronounced impact on *in vivo* anti-tumorgenicity in both subcutaneous and intracranial xenograft models (Wolf et al, under review). Furthermore, HK2 translocation to the mitochondria is regulated by growth factor induced oncogenic signaling pathways, such as increased EGFR and PI3K/AKT activation, known to be aberrant in GBMs [64].

Interestingly, in a recent report comparing the expression profile of primary breast cancers with unlinked brain metastasis, HK2 was found to be consistently up-regulated in the brain metastatic tumors but not the primary breast cancers. This supports that there are important micro-environmental influences perhaps unique to the brain that may be promoting HK2 expression and tumor growth [65].

### PKM2

Pyruvate kinase (PK) is the last irreversible step of glycolysis, catalyzing the reaction of phospho-enolpyruvate (PEP) to pyruvate. Humans contain two PK genes *(PKLR and PKM2*) and four PK isozymes (L, R, M1 and M2). PKM1 and PKM2 are alternatively spliced transcripts of the *PKM2* gene. PKM1 is primarily expressed in the brain and muscle while PKM2 is found in proliferating tissues including embryonic tissue and tumor cells [66]. Cantley and colleagues reported the preferential expression of the embryonic PKM2 splice variant, rather than adult PKM1, in a panel of cancer cell lines [17]. The tumor specific PKM2 oscillates between an active tetramer and a less active dimer, regulated by phosphotyrosine binding, although the details of this regulation have yet to be deciphered [67,68]. The decreased activity of the PKM dimer may result in shunting of upstream glycolytic intermediates into biosynthetic pathway, including lipid synthesis [68]. Furthermore, it is not clear how tumor cells generate lactate if glucose is shuttled into biosynthetic pathways, although anaplerotic reactions may be crucial. PKM2-expressing cells with reduced PK activity may favour the PEP-dependent histidine phosphorylation of the glycolytic enzyme phosphoglycerate mutase (PGAM1), providing an alternative glycolytic pathway favouring anabolic metabolism [69]. Isoform selective inhibition of PKM2 using small molecular inhibitors is reportedly feasible by targeting its unique region for allosteric regulation with limited effects on PKM1 [70]. GBM cells do express high levels of PKM2 but also express PKM1 supporting an incomplete switch in splice isoforms. Knock down of total PKM2 does not favour the switch in GBM metabolism from aerobic glycolysis to OXPHOS, unlike knockdown of HK2 (Wolf et al., under review).

### PDK

Pyruvate dehydrogenase (PDH) is a mitochondrial multi-enzyme complex that catalyzes the oxidative decarboxylation of pyruvate, whose enzymatic activity is regulated by a phosphorylation/dephosphorylation cycle. The mitochondrial matrix protein Pyruvate dehyrogenase kinase (PDK1 to 4) is an important inhibitor of OXPHOS via its phosphorylation of the E1 alpha subunit of PDH. HIF1α transactivates PDK1 resulting in decreased conversion of pyruvate to acetyl-coA and compromising OXPHOS [71]. Treatment of cancer cells with the small molecule inhibitor of PDK dichloroacetate (DCA), currently employed for the treatment of congenital lactic acidosis, was found to activate OXPHOS and promote apoptosis in cancer cells [72]. DCA is believed to sensitize to apoptosis via two mechanisms: 1) enhanced flux of electrons through the ETC resulting in greater depolarization of the mitochondrial membrane, which is generally hyperpolarized in tumor cells, and enhanced release of apoptotic cytochrome c; 2) return in OXPHOS function generating greater ROS, up-regulating voltage-dependent K+ channel leading to an efflux of K+ and activation of caspases [72]. DCA, which crosses the blood-brain barrier, has been administered in a small number of GBM patients with limited toxicity aside from a dose-dependent, reversible peripheral neuropathy [73]. However, large, randomized controlled trials are warranted to ascertain its effectiveness in GBM patients. Furthermore, whether DCA can sensitize GBM cells to temozolomide or radiation remains to be determined.

## THERAPEUTIC TARGETING IN GBM

The metabolic divergence between GBM and normal cells may provide novel therapeutic strategies to be exploited. Increasing tumorigenicity correlates with greater sensitivity to glycolytic inhibitors [74]. As depicted in Figure 1, any number of steps in glucose metabolism may act as putative therapeutic targets, with varying extent of side effects. However, upon screening of TCGA data, only a few enzymes are strongly up-regulated relative to normal brain, which includes HK2.

Selective targeting of HK2 may have dual effects on cells by inhibiting glycolysis and promoting OXPHOS thereby impacting proliferation as well as dissociating the anti-apoptotic interaction between HK2 and VDAC. Current agents targeting HK2 (e.g. 3-bromopyruvate, 2-deoxyglucose) have limited clinical potential in GBMs due to non-specificity (e.g. impact on HK1 or other metabolic proteins) and systemic toxicity. Newer agents are currently under development aimed at interfering more selectively the interaction between HK2 and VDAC (e.g. methyl jasmonate) [75]. However, to date, no specific inhibitor of HK2 has been developed. It also remains to be investigated whether combined targeting of metabolic enzymes, including inhibitors to HK2, PKM2 and DCA for PDK, will further enhance GBM cell death.

Future studies should strive to investigate the impact of inhibition of specific enzymes (e.g. with DCA) on metabolic network as a whole including fatty acid, nucleotide and amino acid metabolism. Metabolomics can assist in the quantification of metabolites in cells, tissues or sera, which may be particularly informative in pathologically heterogeneous human GBM tissues. The metabolomic profile could then be correlated with the transcript or proteomic profile of corresponding tissues, potentially yielding novel diagnostic or prognostic markers. Understanding how these metabolic networks vary and their importance for proliferation and resistance to cell death may identify further targeted therapeutic strategies for GBM patients.

## SUPPLEMENTAL FIGURES


