# Erratum: Targeting Metabolic Remodeling in Glioblastoma Multiforme

**DOI:** 10.18632/oncotarget.26219

**Published:** 2018-10-05

**Authors:** Amparo Wolf, Sameer Agnihotri, Abhijit Guha

**Affiliations:** ^1^ The Arthur and Sonia Labatt Brain Tumor Research Centre, Hospital for Sick Children Research Institute, University of Toronto, Toronto, Ontario, Canada, M5G 1L7; ^2^ Division of Neurosurgery, Toronto Western Hospital, University of Toronto, Toronto, Ontario, Canada, M5T 2S8

**This article has been corrected:** During production, the page numbers for this article were listed incorrectly. The page numbers have now been adjusted to show the proper pagination.

Original article: Oncotarget. 2010; 1:552-562. https://doi.org/10.18632/oncotarget.190

