# Supplementary material for: Targeting Metabolic Remodeling in Glioblastoma Multiforme
Source: Oncotarget. 2010 Oct 27;1(7):552–62. doi: 10.18632/oncotarget.190 (PMC3035636; doi:10.18632/oncotarget.190)
Supplement: Supplementary file 1 [file oncotarget-01-567-s001.pdf]

Table 1: Metabolic related genes differentially expressed in GBM at the RNA level

Up-regulated		Down-regulated	
**Primary Metabolic Process**	**Gene**	**Primary Metabolic Process**	**Gene**
Glucose Metabolism	FABP5<br>HK2<br>ITGB1<br>MSTN<br>MYC<br>PRKAA1<br>ZBTB20<br>IDH1	Glucose Metabolism	ALDH5A1<br>BDNF<br>CACNA1A<br>NISCH<br>NPY1R<br>PDK2<br>PDK4<br>HK1<br>IDH3a
Fatty Acid Metabolism	ACOT9<br>ALOX5<br>ALOX15B<br>ALOX5AP<br>CAV1<br>CD36<br>CD74<br>CEBPB<br>CROT<br>DBI<br>EDN2<br>ELOVL2<br>FABP4<br>FCER1A<br>GGT5<br>LPL<br>PDPN<br>PECI<br>PLA2G5<br>PTGS1<br>QKI<br>SLC27A3<br>SUCLG2<br>SYK<br>TNFRSF1A<br>TNXB	Fatty Acid Metabolism	AACS<br>ACOT7<br>ACSL6<br>AKR1C2<br>ALDH5A1<br>ANGPTL3<br>ANKRD26<br>FAAH<br>HNF4A<br>MYO5A<br>OXCT1<br>PDK4<br>PLP1<br>PRKAR2B<br>SCD<br>SLC27A2<br>SNCA
Glutamine Metabolism	DDAH2<br>GFPT2<br>MECP2	Glutamine Metabolism	ALDH5A1<br>GLS<br>GLUD1<br>GLUD2
Nucleotide Metabolism	ADA<br>REXO2<br>TYMP<br>UPP1	Nucleotide Metabolism	CSGALNACT1<br>ENTPD3<br>UGP2

Supplemental Table: TCGA samples used in analysis

Normal Brain Sample ID	GBM Sample ID
TCGA-06-0673-11	TCGA-06-0192-01
TCGA-06-0675-11	TCGA-06-0649-01
TCGA-06-0676-11	TCGA-06-0686-01
TCGA-06-0678-11	TCGA-06-0743-01
TCGA-06-0680-11	TCGA-06-0744-01
TCGA-06-0681-11	TCGA-06-0745-01
TCGA-08-0623-11	TCGA-06-0747-01
TCGA-08-0625-11	TCGA-06-0749-01
TCGA-08-0626-11	TCGA-06-0750-01
TCGA-08-0627-11	TCGA-12-0654-01
	TCGA-12-0656-01
	TCGA-12-0657-01
	TCGA-12-0688-01
	TCGA-12-0692-01
	TCGA-12-0703-01
	TCGA-12-0707-01
	TCGA-12-0772-01
	TCGA-12-0773-01
	TCGA-12-0775-01
	TCGA-12-0776-01
	TCGA-12-0778-01
	TCGA-12-0780-01
	TCGA-15-0742-01
	TCGA-06-0216-01A
	TCGA-06-0216-01B
